# Antimicrobial resistance and toxigenic profiles of bacteria isolated from tropical shrimps (*Farfantepenaeus notialis* and *Penaeus monodon*) in Cameroun

**DOI:** 10.1186/s13104-020-05184-1

**Published:** 2020-07-29

**Authors:** Sabine Ninelle Nga Ombede, Victorien Dougnon, Hornel Koudokpon, Esther Deguenon, Rajeunie Pernelle Jaelle Mindzie Ngomo, Carine Tchibozo, Jean Pierre Gnimatin, François Tchoumbougnang, Anges Yadouleton, Jacques Dougnon

**Affiliations:** 1 Department of Processing and Quality Control of Fishery Products, Institute of Fisheries and Aquatic Sciences, Douala, Cameroon; 2 Research Unit in Applied Microbiology and Pharmacology of Natural Substances, Polytechnic School of Abomey-Calavi, University of Abomey-Calavi, Abomey-Calavi, Benin; 3 Reference Laboratory of Hemorragic Fevers in Benin, Ministry of Heath, Cotonou, Benin

**Keywords:** Spoilage, Shrimps, *Bacillus*, Resistance genes, Enterotoxins

## Abstract

**Objective:**

Post-harvest shrimp losses are a big problem due to the proliferation of spoilage bacteria. Presence and multiplication of these bacteria promotes the emergence of food-borne diseases. This study was carried out to characterize some spoilage bacteria from tropical brackish water shrimps and black tiger shrimps stored in ambient temperature (25 °C).

**Results:**

22 isolates of *Bacillus* spp; 09 isolates of Coagulase Negative *Staphylococci* (CNS) and 04 isolates of enterobacteria such as *Pantoea* spp (01); *Serratia plymutica* (01) and *Serratia rubidaea* (02) have been identified. Resistance and virulence genes were then detected. All isolates expressed resistance to at least three of antibiotics tested. 03 isolates of enterobacteria were susceptible to cetfazidim and amoxicillin-clavulanic acid. *Bacillus* spp showed total susceptibility to cefixim, ertapenem and cetfazidim. *Staphylococci* were susceptible to clindamycin. *Pantoea* spp was resistant to all antibiotics but exhibited intermediate susceptibility to amoxicillin-clavulanic acid. 04 isolates of *Staphylococci* were positive to mecA resistances genes. All the enterobacteria harbor no tetracycline resistance genes. All the isolates of *Bacillus* exhibited the presence of enterotoxin genes. Also, a high prevalence of 21 isolates to hemolytic enterotoxins was noted. 17 isolates from them kept ability to cell-lyse factor production like sphingomyelinase activities. The majority of *Bacillus* isolates identified by the present study poses a potential risk of food poisoning due to the prevalence of toxin genes found.

## Introduction

Shrimps are great component of global seafood production. In general, they contain good quantities of digestible proteins, essential aminoacids, bioactive peptides, long-chain polyunsaturated fatty acids [1]. In Cameroun, tropical brackish water shrimp (*Farfantepenaeus notialis*) and black tiger shrimp (*Penaeus monodon*) are species widely consumed [2]. Shrimps are generally safe for consumption but their exposure to handling practices may occasionally entail health risks [3, 4]. Many retailers inappropriately stored shrimps in addition to poorly handling practices causing postharvest degradation [5]. Specific spoilage organisms will change characteristics of food as consequence of contamination [6]. Many studies showed emergence of foodborne diseases due to spoilage bacteria [7]. Gram-positive bacteria are slightly dominant in tropical warm water [8]. This suggests their great implications in spoilage, especially at ambient temperature [9]. Foodborne illness could result from the fact that those bacteria harbor enterotoxins and resistance genes [7]. The aim of this study was then to explore antibiotics resistance and virulence factor of spoilage bacteria isolated from shrimps stored in ambient temperature (25 °C) in our markets.

## Main text

### Materials and methods

#### Collection and storage of samples

Experiments were carried out with 5 kg of tropical brackish water shrimp (*Farfantepenaeus notialis*) and the same quantity of black tiger shrimp (*Penaeus monodon*). The sampling took place from November to December 2018. Freshly harvested shrimp were purchased from ‘Youpwe fisheries market’ in Douala, Cameroon. Those samples were transported to the laboratory in iced box. They were then packed into four groups of 250 g portions per species in sterile plastic bags and kept at − 20 °C until lab processing. Every 2 days, one plastic bag from each species was randomly taken for enumeration and isolation of total spoilage bacteria. Before this, shrimps were removed from fridge and left at 25 °C for 24 h in the laboratory. This was done at room temperature because shrimps are sold in the markets without cold conditions and allowed spoilage odor to be produced.

#### Identification of spoilage bacteria

25 grams of shrimp per specie were aseptically added to 225 ml of peptone water and homogenized [10]. Seven fold dilutions of each homogenate were prepared and 0.1 ml of 10^−7^ dilution was used for enumeration and isolation on nutrient agar. Replicate plates were incubated in aerobiosis and in anaerobiosis at 37 °C. Thereafter counting, three characteristic isolates per plate were purified on Mueller Hinton. Pure isolates were stored at − 80 °C in 20% glycerol. Identification of bacterial isolates was then carried out using biochemical tests.

#### Detection of resistance profile

An overnight bacterial pre-culture was diluted to obtain a turbidity of 0.5 McFarland. Kirby Bauer techniques were used to perform susceptibility testing (Antibiogram Committee of the French Society of Microbiology, CA-SFM, 2012). Broth cultures were aseptically swabbed on Mueller Hinton agar. Antibiotic disks were placed on the agar plates and incubated 24 h at 37 °C. Inhibition zone diameters were measured after 24 h. Susceptibility or resistance was determined according to CA-SFM (2012). Antibiotic disks (HIMEDIA, India) used were amoxicillin (30 μg), amoxicillin-clavulanic acid (30 μg), cefixim (5 μg), ceftazidim (30 μg), ceftriazone (30 μg), ciprofoxacin (5 μg), clindamycin (10 μg) colistin (25 μg), ertapenem (10 μg), erythromycin (15 μg) fosfomycin (50 μg), imipenem (10 μg), nalidixic acid (30 μg), oxacillin (1 μg), penicillin (10 μg), pristinamycin (15 μg) and vancomycin (30 μg).

#### Molecular identification of antibiotic resistance genes

DNA was extracted from the overnight colony obtained by spreading on Muller Hinton agar. DNA was extracted from isolates using Qiagen blue extraction kit. PCR mixture of 23 µl contained 10 Nmole of genomic DNA, 0.5 µl of each primer, 0.5 µl dNTP, 2.5 µl Buffer with MgCl2, 0.125 µl Taq DNA Polymerase, and 16 µl distilled water. For enterobacteria and staphylococci isolates, PCR detection was performed for the following different resistance genes : macrolide-resistance genes, vancomycin resistance genes, methicillin resistance genes and tetracycline resistance genes. PCR conditions included initial denaturation for 5 min at 94 °C, followed by 40 cycles of denaturation for 45 s at 94 °C, annealing for 55 s at 45 °C, extension for 90 s at 72 °C and final extension for 10 min at 72 °C. PCR conditions for mecA were in accordance with those described by Igbinosa and Beshiru [11]. For *Bacillus*, virulence and enterotoxins genes were investigated. Genes encoding hemolysin (hbl-D/A), non-hemolytic enterotoxin (nheB), *B. cereus* enterotoxin T (bceT) and enterotoxin FM (entFM) were screened. Additional file 1: Table S1 show the primers used in the study. Virulence factors targeting genes coding for two phospholipases associated with cell lysis, sphingomyelinase (sph) and phosphatidylinositol-specific phospholipase C (piplc) were also investigated in accordance with Matarante et al. [12] and Mohammadou et al. [13]. PCR products were analyzed on 1.5% (w/v) agarose gel stained (120 volts at 35 min) with ethidium bromide (0.5 µg/ml) and visualized by UV. Additional file 1: Table S2 shows the primers of virulence factor genes detection.

### Results

#### Identification of spoilage bacteria

Shrimps contained more than 10^7^ germs/g after 24 h in ambient storage. Additional file 1: Figure S1 shows the microbial population present in tropical shrimps. Results showed the main presence of Gram-positive bacteria such as *Bacillus* spp and Coagulase Negative Staphylococci (CNS). Some isolates of enterobacteria (*Serratia* spp and *Pantoea* spp) were also identified.

#### Resistance profile

All the isolates of Coagulase Negative *Staphylococci* were susceptible to clindamycin. They showed total resistance to oxacillin and penicillin. The isolates were resistant to ciprofloxacin, colistin and vancomycin (55.56%), to nalidixic acid (88.89%). 15 isolates showed resistance to ceftazidim, ceftriaxon, cefixim and amoxicillin. Table 1 presents the antimicrobial susceptibility profile of *Staphylococci*.

**Table 1:** Antimicrobial susceptibility of *Staphylococci*

Antibiotics	S if ≥	R if <	S %	I %	R %
Ciprofloxacin	25	22	3 (33.33%)	1 (11.11%)	5 (5.56%)
Pristinamycin	22	19	5 (55.56%)	1 (11.11%)	3 (33.33%)
Penicillin	29	18	0	0	9 (100%)
Fosfomycin	14	14	6 (66.67%)	0	3 (33.33%)
Erythromycin	22	17	5 (55.56%)	1 (11.11%)	3 (33.33%)
Clindamycin	15	15	9 (100%)	0	0
Nalidixic Acid	20	15	1 (11.11%)	0	8 (88.89%)
Vancomycin	17	–	4 (44.44%)	0	5 (55.56%)
Oxacillin	21	21	0	0	9 (100%)
Colistin	15	15	4 (44.44%)	0	5 (55.56%)

*S* sensible, *R* resistant, *I* intermediate

Enterobacteria showed total resistance to ceftriazon, cefixim, amoxicillin and ertapenem. Only isolates of *Pantoea* spp showed intermediate resistance to amoxicillin—clavulanic acid and total resistance to all other antibiotics tested. *Bacillus* spp were susceptible to imipenem. 59.09% of *Bacillus* spp exhibited intermediate resistance to ciprofloxacin and amoxicillin-clavulanic acid. Table 2 shows antimicrobial susceptibility profile of enterobacteria and *Bacillus* spp.

**Table 2:** Antimicrobial susceptibility of enterobacteria and Bacillus species

Antibiotics	S if≥	R if <	Enterobacteria			*Bacillus* spp		
			S	I	R	S %	I %	R %
Amoxicillin - clavulanic Acid	23	16	3	1	–	8 (36.36%)	13 (59.09%)	1 (4.55%)
Vancomycin	17	–	2	–	2	7 (31.82%)	–	15 (68.18%)
Ceftazidim	21	19	–	1	3	–	–	22 (100%)
Impenem	21	18	3	–	1	22 (100%)	–	–
Ciprofloxacin	25	22	1	2	1	6 (27.27%)	6 (27.27%)	10 (45.45%)
Ceftriazone	26	23			4	0	1 (4.55%)	21 (95.45%)
Cefixim	25	22			4	0	0	22 (100%)
Amoxicillin	23	16			4	0	1 (4.55%)	21 (95.45%)
Ertapenem	28	26			4	0	0	22 (100%)

*S* sensible, *R* resistant, *I* intermediate

#### Resistance and enterotoxins-virulence genes

**Fig. 1:**
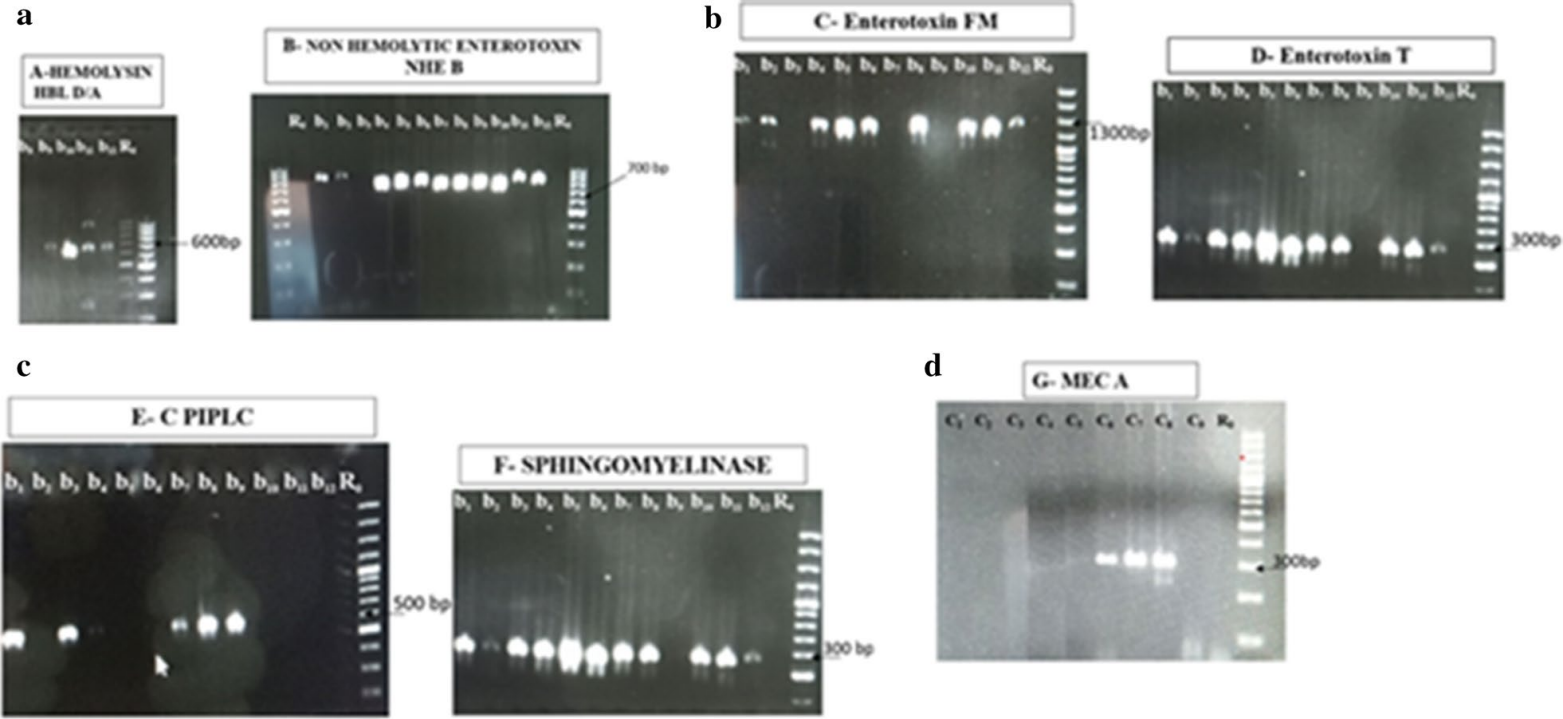
PCR gene migration

*Bacillus* isolates isolated harbor at least two enterotoxins genes. Only one isolate was positive to hemolysin gene. Three isolates were negative to sph and piplo virulence genes. All *Bacillus* isolates (08) harbor *Bacillus cereus* enterotoxin T and also the sph gene. 04 isolates of *Staphylococci* exhibited the presence of MecA resistant gene. All enterobacteria isolates were tested negative to tetracycline resistance gene. Figure 1 shows the results of PCR product migration.

### Discussion

#### Identification of spoilage bacteria

This study was carried out to explore resistance and virulence genes of spoilage bacteria isolated from shrimps in Cameroun. Results of this study are similar to those of Dabadé et al. [9] who mainly found positive Gram bacteria, especially lactic bacteria and enterobacteria in black tropical brackish water shrimp. Those Authors revealed that potentially spoilage bacteria in tropical shrimps in Benin may be H_2_S-producing dominant group of bacteria. Finding in this study suggests that *Bacillus* and *Staphylococci* species are responsible for spoilage detected in tropical shrimp. The results of this study are different from those of Srinivasan and Saranraj [14] who found *Vibrio cholerae, Pseudomonas fluorescens, Salmonella* Typhi*, Staphylococcus aureus* and *Escherichia coli*. These microorganisms are common in polluted water, so it is possible to find them in the seafood. The results obtained are comparable to those of Al Bulushi et al. [8] who who also isolated *Staphylococci* in their study. They also related that the frequency of *Staphylococci* in marine environments depend on geographic location. *Bacillus* species in coastal fisheries are also common, despite the relative abundance of *Staphylococcus* spp [8]. Abundance of *Bacillus* in coastal waters and marine fisheries depends on temperature, depth and decline distance from the coast and, suggests that *Bacillus* species in coastal water originate from runoff [15]. Mohammadou et al. [13] reported *Bacillus* species such as *Bacillus cereus, Bacillus thuringiensis* and *Bacillus anthracis* were well-known as food poisoning in spoilage food. Gram-positive bacteria were more abundant than Gram-negative in tropical water. According to Al bulushi et al. [8], their influence in spoilage remain little probed. Findings of this study also showed low frequency of enterobacteria such as *Serratia* and *Pantoea* isolates who grow in a broad range of temperatures and substrates according to Garrity et al. [16]. Many species related with food spoilage, have been described as opportunistic human pathogens [17–19].

#### Antimicrobial susceptibility profile

The presence of *Pantoea* spp and *Serratia* spp in food is often neglected, when compared to classic multidrug-resistant Gram-negative pathogens [20–22] likewise in this study. They are opportunistic pathogens among the most common causes of nosocomial diseases and transmissible by food ingestion. Resistance diversity of *Bacillus* isolates in the study is not similar to the works of Mohammadou et al. [13]. Those authors revealed relative susceptibility of *Bacillus* isolates to antibiotics unlike our results. Chaves et al. [7] also found susceptibilities of *Bacillus* isolated from Brazilian food to gentamicin and tetracycline. Drug resistance of isolates in this study may be attributed to the spores of *Bacillus* species. Mohammadou et al. [13] found that *B. subtilus* was able to produce bacteriocins such as subtilin in Mbuja.

#### Resistance and enterotoxins-virulence genes

Tetracycline resistance genes can be explored to current and emerging multidrug-resistant pathogens including carbapenem-resistant Enterobacteriaceae. None enterobacteria presented tetracycline resistance genes, although they were all resistant to ertapenem. MecA genes as presented in this study about *Staphylococci* are similar to those of Ali [23] and Igbinosa and Beshiru [11]. MecA gene is present in all *Staphylococci* isolates and is known to encode penicillin binding protein 2a (PBP2a). Beta-lactam resistance is mostly attributed to mutations in mecA gene, but other genetic elements may also be considered for the explanation of the mechanism of resistance. Half of *Bacillus* isolates were positive to HBL genes. These results are similar to those of Chaves et al. [7]. The same authors demonstrated prevalence of hemolytic toxins in *Bacillus* isolates from fermented foods. Many authors correlate prevalence of enterotoxins HBL-NHE [24]. According to Trans et al. [25], wall peptidase (sph genes and PIPLC genes) are involved in adhesion, biofilm formation and virulence. Prevalence of entFM genes in more than half of *Bacillus* isolates is located on the chromosome and appears to be common to *Bacillus thuringiensis* and *B. cereus*. Detection of EntFM is similar to the results of Mohammadou et al. [13]. Presence of Bcet gene is evidence of presence of *B. cereus* among isolates. Agata et al. [26] also demonstrated that prevalence of this gene carried out other isolates than *B. cereus*. The majority of the isolates isolated pose a potential risk of food poisoning due to their antibiotic resistance profile and the prevalence of toxin genes found in *Bacillus* isolates. The surveillance of the quality control of those seafoods should be enhanced in the country, from the water to the final sellers. This is important and urgent to protect consumers’ health.

## Limitations

To better understand the links between spoilage and the bacteria involved, it would be useful to identify isolates using advanced methods as 16S rRNA gene sequencing or species-specific PCR. The strains will be kept at − 20 °C for sequencing in Helsinki (Finland).

## **Supplementary information**

**Additional file 1: Table S1.** Primers of resistance genes. **Table S2.** Primers of virulence genes. **Figure S1.** Microbial population present in tropical shrimps.

## Data Availability

All data generated or analyzed during this study are included in this published article and additional files.

## Abbreviation

CNS: Coagulase Negative *Staphylococci*
