# Supplementary material for: Antimicrobial resistance and toxigenic profiles of bacteria isolated from tropical shrimps (Farfantepenaeus notialis and Penaeus monodon) in Cameroun
Source: BMC Res Notes. 2020 Jul 29;13:358. doi: 10.1186/s13104-020-05184-1 (PMC7391528; doi:10.1186/s13104-020-05184-1)
Supplement: Supplementary file 1 — Additional file 1: Table S1. Primers of resistance genes. Table S2. Primers of virulence genes. Figure S1. Microbial population present in tropical shrimps. [file 13104_2020_5184_MOESM1_ESM.docx]

**Table S1: Primers of resistance genes**

| **Bacteria strains** | **Genes** | **Primers** | **Primer sequence (5′-3′)** | **Size (bp)** | **References** |
| --- | --- | --- | --- | --- | --- |
| Enterobacteria | tet(B) | BF  BR | CAG TGC TGT TGT TGT CAT TAA  GCT TGG AAT ACT GAG TGT AA | 571 | Miranda et al., 2003 |
|  | tet(C) | CF  CR | TTG CAT GCA CCA TTC CTT GCG  ATG GTC GTC ATC TAC CTG CC | 522 | Miranda et al., 2003 |
|  | tet(D) | DF  DR | GGA TAT CTC ACC GCA TCT GC  CAT CCA TCC GGA AGT GAT AGC | 436 | Miranda et al., 2003 |
|  | tet(E) | EF  ER | TCC ATA CGC GAG ATG ATC TCC  CGA TTA CAG CTG TCA GGT GGG | 442 | Miranda et al., 2003 |
|  | tet(G) | GF  GR | GCT GGA TGA TGC ATT GCG CG  ATG GTC TGC GTA GTA TTG GC | 554 | Soge et al.,2009 |
|  | tet(M/O/S) | M4  M6 | GAA GCC CAG AAA GGA TTY GGT  GTT TAT CAC GGA AGY GCW A | 686 | Miranda et al., 2003 |
| *Staphylococcus spp* | mecA | mecA1  mecA2 | GTAG AAAT GACT GAAC GTCC GATAA CCAA TTCC ACAT TGTT TCGG TCTAA | 310 | Geha et al.1994 |
|  | vanA | van A1  van A2 | GGGAAAACGACAATTGC  GTACAATGCGGCCGTTA | 732 | Dutka-Malen et al., 1995 |
|  | vanB | van B1  van B2 | GTGC TGCG AGAT ACCA CAGA  CGAACACCATGCAACATTTC | 1145 | Ramos-Trujillo et al., 2003 |
|  | mphC | mph (C)-1 mph (C)-2 | GAGA CTAC CAAG AAGA CCTGACG CATA CGCC GATT CTCC TGAT | 722 | Lüthje and Schwarz, 2006 |
|  | ermA | erm(A)-1 erm(A)-2 | GCGGTAAACCCCTCTGAG GCCTGTCGGAATTGG | 434 | Werckenthin and Schwarz., 2000 |
|  | ermB | erm(B)-1 erm(B)-2 | CATT TAAC GACG AAAC TGGC GGAA CATC TGTG GTAT GGCG | 425 | Jensen et al.,1999 |
|  | ermC | erm(C)-1 erm(C)-2 | ATCT TTGA AATC GGCT CAGG  CAAA CCCG TATT CCAC GATT | 295 | Jensen et al., 1999 |

**Table S2: Primers of virulence genes**

| Reference | Primer name | Primer sequence (5’ - 3’) | Amplicon size (bp) | | Reference |
| --- | --- | --- | --- | --- | --- |
| Hbl-D/A | hblD-f  hblA-r | GGAGCGGTCGTTATTGTTGT  GCCGTATCTCCATTGTTCGT | 623 | | Matarante et al., 2004 |
| nheB | nheB 1500S  nheB 2269A | CTATCAGCACTTATGGCAG | 769 | | Granum et al., 1999 |
|  |  | ACTCCTAGCGGTGTTCC |  |  |  |
| bceT | ETF | TTACATTACCAGGACGTGCTT | 428 | | Agata et al., 1995 |
|  | ETR | TGTTTGTGATTGTAATTCAGG |  |  |  |
| entFM | EntA | ATGAAAAAAGTAATTTGCAGG | 1269 | | Asano et al.,1997 |
|  | EntA | TTAGTATGCTTTTGTGTAACC |  |  |  |
| Sph | Ph1 | CGTGCCGATTTAATTGGGGC | 558 | | Hisieh et al., 1999 |
|  | Ph2 | CAATGTTTTAAACATGGATGCG |  |  |  |
| Piplc | PC105 PC106 | CGCTATCAATGGACCATGG  GGACTATTCCATGCTGTACC | 569 | Damgaard et al.,1996 | |

**Figure S1: Microbial population present in tropical shrimps**
